# Mean systolic blood pressure upon admission and clinical outcomes after endovascular treatment in patients with large vessel occlusion beyond 24 hours

**DOI:** 10.3389/fneur.2026.1789389

**Published:** 2026-04-21

**Authors:** Feixue Yue, Haiqi Li, Kaili Chen, Mingchao Shi, Hengyu Ji, Jinting He, Shouchun Wang

**Affiliations:** 1 Department of Neurology, China-Japan Union Hospital of Jilin University, Changchun, China; 2 Department of Neurology, First Hospital of Jilin University, Changchun, China

**Keywords:** anterior circulation large vessel occlusion, clinical outcomes, endovascular treatment, ischemic stroke, mean systolic blood pressure

## Abstract

**Background and purpose:**

Recently, several clinical studies have shown that higher systolic blood pressure (SBP) upon admission is associated with poor clinical outcomes in patients with ischemic stroke following endovascular treatment (EVT). However, the effect of the mean systolic blood pressure (MSBP) upon admission on patients with acute anterior circulation large vessel occlusion (ACLVO) who underwent EVT after 24 h is unclear. We sought to assess the association between MSBP at admission and clinical outcomes in patients treated with EVT for acute ACLVO beyond 24 h after onset.

**Methods:**

Patients with ACLVO treated with EVT more than 24 h after symptom onset were consecutively enrolled during a 3-year period at a tertiary stroke center. MSBP was measured at admission using an automatic cuff recording. The efficacy outcome was a 3-month good functional outcome (modified Rankin Scale score of 0–2). Symptomatic intracerebral hemorrhage (sICH) and mortality at 3 months were the safety outcomes.

**Results:**

A total of 57 patients with acute ischemic stroke who met the inclusion criteria were included in the study (mean age 56 ± 12 years, mean National Institute of Health Stroke Scale score 11 ± 5). In the Firth penalized likelihood logistic regression analyses adjusted for confounding factors, higher MSBP levels were significantly negatively correlated with good clinical outcomes (odds ratio: 0.012; 95% confidence interval: 0.000–0.204). There was no significant association between high SBP and sICH or mortality.

**Conclusion:**

Our study suggests that higher MSBP at admission is an independent predictor of a poor clinical outcome at 3 months in patients treated with EVT for acute ACLVO beyond 24 h after onset.

## 1 Introduction

The efficacy of endovascular treatment (EVT) in patients with acute anterior circulation large vessel occlusion (ACLVO) is well-established (1). For such patients, the acute phase of ischemic stroke is mostly characterized by high blood pressure (2, 3). This may be a compensatory mechanism that increases blood perfusion to the ischemic regions of the brain (4). The impaired dynamic regulation of cerebral blood flow in the cerebral hemispheres following ischemic stroke can make early blood pressure control more challenging (5). Recent studies have shown that higher systolic blood pressure (SBP) upon admission is significantly associated with a poor prognosis after thrombectomy for ACLVO (6–8). However, the effect of SBP on ischemic stroke may differ among populations and stroke subtypes.

Previous studies have shown that EVT beyond 24 h that meets the criteria of the DAWN trial or the DEFUSE-3 trial is safe and effective (9, 10). A predominantly Asian study reached a similar conclusion (11). However, there is still a lack of understanding of the factors related to the prognosis of patients with acute ACLVO who undergo EVT after 24 h. Furthermore, whether MSBP affects the prognosis of patients with this subtype remains unclear. To our knowledge, this association has not been examined in patients with acute anterior circulatory EVT beyond 24 h.

Therefore, we aimed to analyze the association between MSBP and clinical outcomes in patients with acute ACLVO who underwent EVT after 24 h.

## 2 Materials and methods

### 2.1 Study population

**Figure 1:**
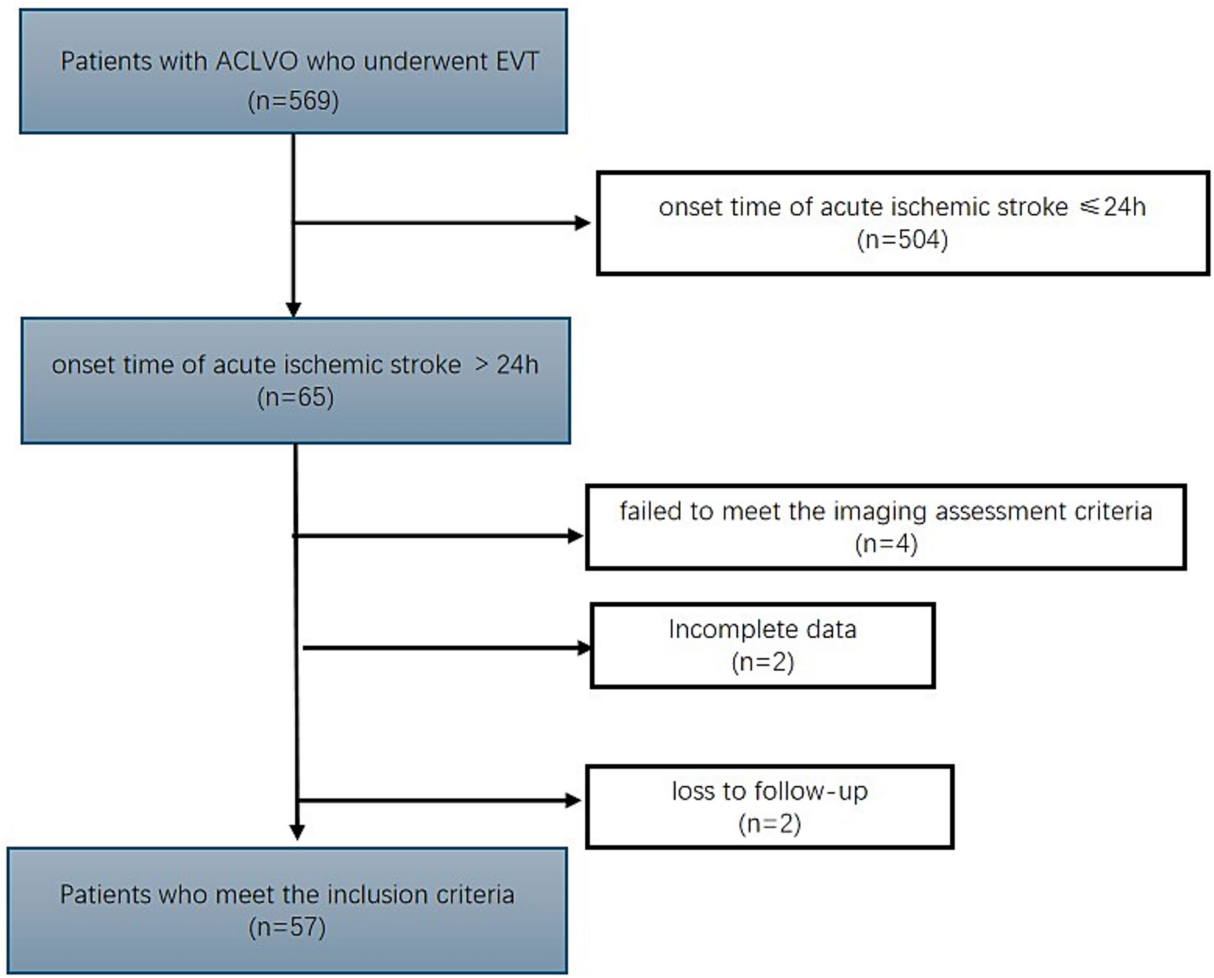
Flowchart of patient selection for ACLVO treated with EVT beyond 24 h after symptom onset.

This retrospective study focused on patients with acute ACLVO in the anterior circulation who underwent endovascular treatment beyond 24 h at a high-flow stroke center in China between 2021 and 2024. This study has received ethical approval from the Ethics Committee of Jilin University (approval number 22 K047). Subjects were patients who met the following inclusion criteria: (1) age ⩾18 years; (2) onset time of acute ischemic stroke (AIS) >24 h; (3) ACLVO (internal carotid artery or middle [M1/M2] cerebral artery) confirmed by magnetic resonance angiography (MRA), computed tomographic angiography (CTA), or digital subtraction angiography (DSA); (4) EVT performed beyond 24 h of estimated time of ACLVO; (5) computed tomography perfusion imaging (CTP) showed an initial infarct core volume <50 mL, a volume ratio of ischemic tissue to the infarct core ≥1.8, and a potentially reversible ischemia (penumbra) volume ≥15 mL; and (6) MSBP data at admission is available. Patients were excluded if they: (1) were confirmed to have intracerebral hemorrhage by neuroimaging, (2) had a premorbid modified Rankin Scale (mRS) score >2, (3) had incomplete clinical data, or (4) had a serious terminal illness or were pregnant or lactating. The study flowchart detailing patient enrollment is presented in Figure 1.

### 2.2 Treatments

Eligible patients underwent EVT with stent retrieval, stenting, thrombus aspiration, balloon angioplasty, intra-arterial thrombolysis, or a combination of these. Antiplatelet and anticoagulant drugs were administered according to the guidelines for the management of AIS (12).

### 2.3 Data collection

The following data were recorded for analysis: baseline information, risk factors for stroke, laboratory data, and EVT characteristics, as shown in Tables 1, 2.

**Table 1:** Baseline Clinical Characteristics according to MSBP tertiles.

Baseline Clinical Characteristics	Tertile 1 (*n* = 19)	Tertile 2 (*n* = 18)	Tertile 3 (*n* = 20)	*p*-value*
Age, y; mean ± SD	54.1 ± 9.5	53.9 ± 14.5	60.6 ± 10.5	0.354
Men, *n* (%)	14 (73.7)	14 (73.7)	16 (80.0)	0.037
History of risk factors, *n* (%)				
Hypertension	8 (42.1)	11 (61.1)	18 (90.0)	0.005
DM	6 (31.6)	3 (16.7)	9 (45.0)	0.463
Atrial fibrillation	0 (0.0)	0 (0.0)	1 (5.0)	0.407
Smoking	13 (68.4)	8 (44.4)	8 (40.0)	0.483
Drinking	10 (52.6)	9 (50.0)	11 (55.0)	0.550
Admission serum glucose, mmol/L	7.8 ± 3.6	8.2 ± 3.0	7.5 ± 2.3	0.690
TOAST classification, *n* (%)				0.096
LAA	15 (78.9)	12 (66.7)	14 (73.8)	
CE	0 (0.0)	0 (0.0)	0 (0.0)	
SOE	1 (5.3)	1 (5.6)	1 (5.0)	
SUE	3 (15.8)	5 (27.8)	5 (25.0)	
Initial NIHSS	9.6 ± 4.0	9.6 ± 3.3	13.2 ± 5.3	0.166
Initial ASPECTS	8.5 ± 1.3	8.5 ± 1.6	8.4 ± 1.8	0.194

ASPECTS, Alberta Stroke Program Early CT Score; CE indicates cardioembolism; DM, diabetes mellitus; HT, hypertension; LAA, large artery atherosclerosis; NIHSS, National Institutes of Health Stroke Scale; TOAST, Trial of ORG 10172 in Acute Stroke Treatment; SOE/SUE, stroke of other determined cause /stroke of undetermined cause.

^*^Continuous variables were compared between groups using Student *t* tests. The χ^2^ test was used for noncontinuous variables.

**Table 2:** Procedural-related characteristics according to MSBP tertiles.

Procedural-related characteristics	Tertile 1 (*n* = 19)	Tertile 2 (*n* = 18)	Tertile 3 (*n* = 20)	*p*-value
Occlusion Site, *n* (%)				0.488
C1	6 (31.6)	7 (38.9)	5 (25.0)	
C2-C7	5 (26.3)	4 (22.2)	5 (25.0)	
M1	8 (41.2)	7 (38.9)	9 (45.0)	
M2	0 (0.0)	0 (0.0)	1 (5.0)	
eTICI, *n* (%)				0.640
eTICI 0-2a	0 (0.0)	6 (33.3)	3 (15.0)	
eTICI 2b-3*	19 (100.0)	12 (66.7)	17 (85.0)	
Anesthesia, *n* (%)				0.344
General	8 (42.1)	11 (61.1)	15 (75.0)	
Local	11 (57.9)	7 (38.9)	5 (25.0)	
ASITN/SIR, *n* (%)				0.462
ASITN/SIR0–1	3 (15.8)	4 (22.2)	3 (15.0)	
ASITN/SIR2–4^†^	16 (84.2)	14 (77.8)	17 (85.0)	
Onset to puncture time (min)	2999.0 (1921.0–4559.0)	2110.0 (1614.8–4640.0)	2765.0 (1836.0–5361.8)	0.255
Puncture to recanalization time (min)	70.0 (59.0–100.0)	85.0 (63.8–112.5)	92.0 (67.3–121.5)	0.216

ASITN/SIR, American Society of Interventional and Therapeutic Neuroradiology/Society of Interventional Radiology; C1\C2–C7\M1\M2 indicate C1 or C2–C7 segment of internal carotid\M1 or M2 segment of middle cerebral artery. eTICI, expanded Thrombolysis in Cerebral Infarction.

^*^eTICI score of 2b-3 indicates complete recanalization.

^†^An ASITN/SIR score of 2–4 indicates favorable collateral compensation.

MSBP was measured by trained nurses or physicians upon admission. Patients were divided into three groups according to tertiles of MSBP levels, including the bottom tertile (MSBP <140 mmHg), middle tertile (140 mmHg≤ MSBP <160 mmHg), and the top tertile (MSBP ≥160 mmHg). Successful recanalization after EVT was defined as an expanded treatment for cerebral infarction (eTICI) score of 2b or 3. Non-contrast computed tomography (CT) was used to identify intracerebral hemorrhage. Trained physicians evaluated the relevant imaging data.

### 2.4 Outcome measurements

The clinical outcome measure was the functional outcome at 90 days according to the mRS, and a mRS score between 0 and 2 was defined as a good functional outcome. Mortality at 90 days and symptomatic intracerebral hemorrhage (sICH) after EVT were considered as the safety clinical outcomes. sICH was defined as a newly observed intracranial hemorrhage resulting in an increase of 4 points in the National Institutes of Health Stroke Scale (NIHSS) score before exacerbation or an increase of 2 points in one category.

### 2.5 Statistical analysis

Continuous variables are described as median (interquartile range) or mean ± standard deviation (SD), as appropriate. Categorical variables are presented as proportions. Differences between groups were tested using the *t*-test or Fisher’s exact test for continuous variables and the χ^2^ test for categorical variables. Statistical significance was set at *p* < 0.05.

Univariate logistic regression analysis was performed to determine factors that had a significant effect on clinical outcomes. MSBP was categorized into tertiles, with the lowest tertile designated as the reference group. A binary logistic regression model was constructed to estimate odds ratios (ORs) and their corresponding 95% confidence intervals (CIs) for the clinical outcomes, comparing the middle and highest MSBP tertiles relative to the reference group. To address potential issues of small sample size and complete or quasi-complete separation, a Firth penalized likelihood logistic regression was employed to reduce small-sample bias and mitigate separation, yielding more stable estimates of adjusted ORs and 95% CIs. All covariates with an unadjusted *p*-value of 0.1 or established clinical relevance were included in the multivariate model. Multicollinearity among predictors was assessed using variance inflation factors (VIFs) calculated from a linear regression framework; variables with VIF > 5 were considered to exhibit concerning collinearity. Data analyses were performed using SPSS (version 22.0; IBM Corp., Armonk, NY, United States) and R (version 3.6.3; R Foundation for Statistical Computing, Vienna, Austria).

## 3 Results

### 3.1 Patient characteristics

A total of 569 patients with ACLVO who met the imaging criteria and underwent EVT were consecutively enrolled, of whom 57 met the inclusion criteria. There were 19 patients in the bottom MSBP tertile, 18 patients in the middle MSBP tertile, and 20 patients in the top MSBP tertile. All patients were followed-up for 3 months after EVT. The proportion of male patients was higher than that of female patients in each group. Intravenous thrombolysis was performed before EVT in only five patients (8.7%). The Initial NIHSS scores in the top MSBP tertile were higher than those in the other tertiles. The initial APECTS scores were similar among the three groups. Table 1 presents the baseline clinical and biochemical characteristics according to MSBP levels.

### 3.2 Characteristics of endovascular procedures

Middle cerebral artery (M1\M2) occlusion was found in eight patients (41.2%) in the bottom MSBP tertile, seven patients (38.9%) in the middle MSBP tertile, and 10 patients (50.0%) in the top MSBP tertile. Successful reperfusion (eTICI 2b or 3) was achieved in 48 patients (84.2%). Poor collateral circulation (American Society of Interventional and Therapeutic Neuroradiology/Society of Interventional Radiology, ASITN/SIR 0–1) was found in three patients (15.8%) in the bottom MSBP tertile, four patients (22.2%) in the middle MSBP tertile, and three patients (15.0%) in the top MSBP tertile. The median time from puncture to recanalization in the hypertension group (top MSBP tertile) was 92 (67.3–121.5) min, which was higher than that in the other MSBP groups. Table 2 presents the procedural characteristics of the study.

### 3.3 Clinical outcomes

**Figure 2:**
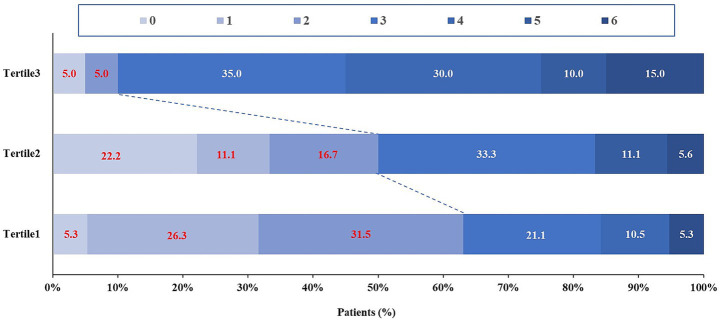
Functional outcomes according to MSBP tertiles. Distribution of modified Rankin Scale (mRS) scores at 90 days in patients treated with endovascular treatment.

There were 23 patients (40.4%) who had a good outcome (mRS of 0 to 2), and 40 patients (70.2%) had an mRS score of 0 to 3 (indicating the ability to walk without assistance). The unadjusted OR and 95% CI for a good shift in the distribution of the mRS score was 0.065 (0.011–0.367) (*p* = 0.002) across baseline MSBP tertiles (Figure 2). Multicollinearity among the predictors was assessed using variance inflation factors (VIFs). All VIF values were below the conventional threshold of 5 (range: 1.089–1.582), indicating no evidence of significant multicollinearity in the model. After adjusting for the confounding factors, in the top MSBP tertile, good clinical outcomes were found to be significantly negatively correlated (OR: 0.012; 95% CI: 0.000–0.204; *p* < 0.001; Table 3) compared with the bottom MSBP tertile. In the multivariate analysis, we found no correlation between the top MSBP tertile and sICH or mortality.

**Table 3:** Association between top MSBP tertiles and outcomes.

Clinical outcomes	Tertile 1 (*n* = 19), *n* (%)	Adjusted OR* (95% CI)	Tertile 2 (*n* = 18), *n* (%)	Adjusted OR* (95% CI)	Tertile 3 (*n* = 20), *n* (%)	Adjusted OR* (95% CI)	*p* Value^†^
Efficacy outcomes at 3-month							
Good outcome (mRS, 0–2)	12 (63.2)	1.00 (Ref)	9 (50.0)	0.438 (0.047–0.311)	2(10.0)	0.012 (0.000–0.204)	<0.001
Safety outcomes							
Symptomatic intracranial hemorrhage	1 (5.3)	1.00 (Ref)	2 (11.1)	1.079 (0.057–17.214)	1 (5.0)	0.716 (0.040–13.820)	0.812
3-month mortality	1 (5.3)	1.00 (Ref)	1 (5.6)	1.040 (0.015–60.470)	3 (15.0)	3.899 (0.144–1298.800)	0.425

^*^The Firth’s penalized likelihood logistic regression test was used to analyze ORs. Adjusted variables: age, sex, initial NIHSS, TOAST, HT, intravenous thrombolysis, recanalization,puncture to recanalization time.

^†^A *p*-value <0.05 as a threshold for statistical significance.

## 4 Discussion

To the best of our knowledge, this cohort study is the first to assess the association between MSBP and functional outcomes among patients with ACLVO treated with EVT beyond 24 h after onset. Our data indicated that the higher baseline MSBP levels (≥160 mmHg) were associated with a poor functional outcome at 3 months. After adjusting for confounding factors, this association remained statistically significant.

In the subsequent analysis of DEFUSE-3, approximately 20% of patients with occlusion of the internal carotid artery or middle cerebral artery had a favorable hypoperfusion ratio, even after more than 24 h of onset; EVT may prevent delayed infarct expansion in such patients (10). Another multicenter study showed that DAWN-eligible patients with occlusion of a large vessel more than 24 h after onset had no significant differences in the favorable outcome (mRS 0–2, 43% vs. 48%, *p* = 0.68), intracranial hemorrhage (5% vs. 6%, *p* = 0.87), or reperfusion rates (mTICI2b–3, 81% vs. 84%, *p* = 0.72) compared with the DAWN trial (9). A study from Korea also showed that EVT treatment could significantly improve the prognosis of such patients (mRS 0–2, OR: 11.08, 95% CI: 1.88–108.60) (11). The favorable prognosis observed in this study is consistent with the above findings. However, we have limited knowledge of the relevant risk factors affecting good prognosis. MSBP upon admission is a risk factor that can be easily measured and regulated.

In recent years, several studies have shown that SBP at admission is significantly associated with poor prognosis after EVT in patients with AIS (7, 8, 13, 14). Similar results were obtained in this study. However, our findings further highlighted the effect of a higher MSBP on ACLVO treated with EVT beyond 24 h. Several mechanisms may explain the potential association between SBP at admission and the clinical outcomes in patients receiving EVT. A larger thrombus burden may have contributed to elevated SBP on admission, and the association between higher SBP and poor prognosis may reflect the underlying thrombus burden (15). In contrast, clinical studies have shown that elevated blood pressure immediately after stroke increases brain edema and blood–brain permeability (16), which may lead to worse clinical outcomes. Another hypothesis is that an acute elevation of SBP creates greater hemodynamics, and compression of the thrombus by flow makes thrombectomy more difficult (17). Furthermore, animal experiments have shown that acute blood pressure elevation in AIS may impair endogenous fibrinolytic capacity, thereby reducing vascular recanalization and adversely affecting prognosis (18, 19). Additionally, an elevated SBP on admission, as a compensatory mechanism, may be an important indicator of severe cerebral ischemia before EVT (13). However, further mechanisms need to be verified in future studies.

In our study, we did not find an increasing trend in the occurrence of sICH among patients with a higher MSBP at admission. This observation is consistent with those of previous studies (3, 14, 20). However, in previous studies, a correlation between higher baseline SBP and a higher sICH risk was found in patients treated with EVT or intravenous alteplase (21, 22). This discrepancy may be due to the lower rate of sICH (7.0%) in our study population. A previous study on patients with AIS treated with EVT showed that a higher SBP at admission was associated with increased 90-day mortality (7, 8, 14). However, the results of recent studies on the correlation between SBP and mortality in patients with ischemic stroke are controversial. Specifically, some findings have suggested no correlation (13, 23), while others have shown a detrimental effect in patients with a higher SBP (7, 8). In the present study, we did not find that a higher MSBP was a significant predictive factor for 90-day mortality in patients with ACLVO after 24 h of treatment with EVT. The differences in the study populations may explain this discrepancy. Further research is required to confirm this association.

Although this study emphasizes the adverse effects of excessively high preoperative MSBP, it must be emphasized that rapidly and abruptly lowering preoperative blood pressure does not benefit the outcome of thrombectomy. On the contrary, excessive hypotension before surgery appears to be harmful. Analysis from the MR CLEAN Registry indicates that there may be a U-shaped relationship between SBP and functional outcomes, meaning that SBP lower than 120 mmHg may significantly increase the risk of adverse prognosis (21). Subsequently, a randomized controlled trial, INTERACT4 (24) (Intensive Ambulance-Delivered Blood-Pressure Reduction in Hyperacute Stroke), also reached a similar conclusion, suggesting that intensive blood pressure lowering increases the risk of adverse events, which may be mediated by impaired collateral perfusion. Additionally, another study has proposed a J-shaped association between preoperative SBP and prognosis, with 150 mmHg as the inflection point. The relatively high SBP is not only independently associated with a poorer clinical prognosis, but also seems to be negatively correlated with the success rate of reperfusion (8). Our study did not identify any obvious U-shaped or J-shaped associations. This discrepancy may be attributed to differences in patient selection and the limited sample size. Moreover, blood pressure variability (BPV) has been consistently demonstrated to be a key predictor of prognosis after thrombectomy. An increase in BPV significantly elevates the risk of adverse outcomes (14, 25–27). Mechanistically, increased blood pressure fluctuations may exacerbate blood–brain barrier disruption, rendering patients more susceptible to hemorrhagic transformation (25). Additionally, blood pressure fluctuations may also induce secondary brain injury by promoting infarct expansion after reperfusion (27). These insights emphasize that in this patient population, attention should be paid not only to the control of excessive hypertension but also to the stability of blood pressure control. Future research should aim to elucidate the specific role of BPV in this patient group.

This study had several limitations. First, we did not assess the effect of other blood pressure variables, such as mean arterial pressure, pulse pressure, and blood pressure variability, on prognosis. Second, because of the current difficulty in recruiting such patients, the correlation between MSBP and other prognostic factors may be indirectly affected. However, our research already presented a larger sample size for data collection in the real world. Furthermore, as this was an observational study, we could not establish a cause–effect relationship between MSBP at admission and clinical outcomes. Additionally, although we adjusted for confounding factors in the multivariate analysis, unmeasured confounding factors may still be present.

In summary, our findings indicate that a higher MSBP may be an independent predictor of unfavorable functional outcomes at 3 months in patients with ACLVO who underwent EVT beyond 24 h. These observations may help physicians to identify patients with ACLVO beyond 24 h after onset who might benefit more or less from EVT. This preliminary finding should be further validated in well-designed prospective multicenter randomized trials.

## Data Availability

The raw data supporting the conclusions of this article will be made available by the authors, without undue reservation.
